# No socioeconomic disparities in the availability of personal care assistance: a population-based cohort analysis of children living with respiratory support

**DOI:** 10.1186/s12939-025-02511-5

**Published:** 2025-05-19

**Authors:** Johan Florén, Åsa Israelsson-Skogsberg, Magnus Ekström, Berit Lindahl, Agneta Markström, Andreas Palm

**Affiliations:** 1 Faculty of Caring Science, University of Borås, Borås, 501 90 Sweden; 2 Department of Clinical Sciences, Division of Respiratory Medicine & Allergology, Lund University, Lund, Sweden; 3 Department of Medical Sciences, Lung- allergy- and sleep research, Karolinska University Hospital, Stockholm, Sweden; 4 Department of Medical Sciences, Respiratory, Allergy and Sleep Research, Uppsala University, Uppsala, Sweden

**Keywords:** Social inequalities, Respiratory insufficiency, HMV, Chronic disease, CPAP, NIV

## Abstract

**Background:**

Children aged 0–18 years who need long-term respiratory support rely on medical technology and comprehensive medical care. For this care to be provided at home, access to medical and social support and care is essential. In Sweden, the most notable form is personal care assistance (PCA), which is granted based on legislation and individual authority decisions. We aim to explore the impact of socioeconomic factors on the availability of PCAs in children on long-term respiratory support.

**Methods:**

This was a retrospective, population-based cohort analysis of children living with respiratory support in the Swedish Quality Registry for Respiratory Failure (Swedevox) between 2015 and 2021, with crosslinked national registry data on socioeconomic factors and PCA. Associations between socioeconomic factors (country of origin, disposable household income, parents’ educational level and marital status) and having been granted PCA were analysed using multivariable regression models.

**Results:**

Of the 600 included children (mean age 5.4 ± 5.1 years), 171 (29%) were granted PCA for a median 235 h/month (interquartile range 56–453). No associations were found between socioeconomic factors and the likelihood of children receiving PCA. Specifically, family income (tertile 2: OR 1.02, 95% CI 0.6–1.7; tertile 3: OR 0.89, 95% CI 0.5–1.5), parental education level (OR 1.08, 95% CI 0.7–1.6), parents’ marital status (OR 0.91, 95% CI 0.5–1.6), and country of origin (OR 1.33, 95% CI 0.9–2.0) were not associated with PCA receipt.

**Conclusion:**

Among children on long-term respiratory support, 29% were granted PCA, which was not associated with their socioeconomic status. While this suggests that care is provided based on need, the low proportion of children granted PCA raises concerns about whether those judged ineligible receive adequate and equitable support.

## Background

Children requiring long-term respiratory support at home have a wide range of underlying conditions that require respiratory intervention. Many of these children face multiple health challenges, requiring complex care from both family and professionals [1–4]. Respiratory support includes continuous positive airway pressure (CPAP); long-term mechanical ventilation (LTMV), tracheal cannula only; high-flow oxygen therapy (HFOT); and phrenic nerve pacing [5]. The respiratory support, along with comprehensive medical care, allows these children to manage conditions that were once life-threatening while maintaining active and healthy lifestyles, avoiding respiratory infections, increasing energy levels and enabling them to engage in societal activities [1, 6–10]. In recent decades, the number of children worldwide who require respiratory support has grown substantially due to improvements in quality and availability of care and respiratory support technology [1, 11, 12]. In Sweden, at least 540 children were using respiratory support at the beginning of 2024, according to Swedevox, with the number growing by approximately 50 children per year between 2015 and 2023 [13].

In Sweden, the Swedish Act concerning Support and Service for Persons with Certain Functional Impairments (LSS) [14] aims to ensure that people with long-lasting physical or mental disabilities have access to a good standard of living and individually tailored personal care assistance (PCA) as well as respite care alleviating the family if needed [14, 15]. Eligibility for PCA under the LSS act is restricted to individuals who belong to one of three specific groups: (1) persons with intellectual disabilities, autism, or conditions resembling autism; (2) persons with significant and permanent intellectual impairments resulting from brain injury in adulthood; or (3) persons with other lasting physical or mental impairments that are not related to normal aging. To qualify for PCA, the individual must be under the age of 65, have a substantial and permanent functional impairment that causes major difficulties in managing basic daily needs, such as personal hygiene, meals, communication, and other essential personal care, and must require extensive, regular support. PCA can range from a few hours of assistance to round-the-clock assistance by multiple carers to enable daily life participation in society and alleviate a family from a burdensome caring situation. The PCA is approved and financed by the municipality and the Swedish Social Insurance Agency [16, 17]. The care given from PCA is described as crucial for those in need of it and a prerequisite to allow a good life involved in society for many children living with long-term respiratory support [6, 17].

However, decisions regarding granting PCA and the number of support hours are often subject to negotiation between the patient/the parents, the municipal authorities/assessment officer, and the Swedish Social Insurance Agency [18, 19]. In recent decades, the trend is towards a decreasing approval rate regarding PCA in general, requiring more comprehensive needs to be granted to it [15, 20, 21]. This decreasing approval rate led to a change in Swedish legislation in 2019, acknowledging breathing as a basic need [14, 22]. Recognising breathing as a basic need has allowed examples such as tracheostomy care and assistance with secretion mobilisation to be included as valid reasons for PCA. This doubled the approval rate for applications and greatly increased the monthly allocation of PCA hours, mostly affecting children aged 0–6 years [23].

There is a well-established relation between a family’s socioeconomic status and mortality and health as well as the quality of life for both healthy children and those living with long-term illnesses [24–28]. Olin, Dunér and Rauch [15] suggests that lower education decreases access to PCA, while other studies [29, 30] describe how parents and young people must actively advocate to secure rightful access to care and support.

### Objectives

With adequate care, children requiring long-term respiratory support can experience life just as healthy and fulfilling as their peers [6]. However, the quality of life for these children and their families is profoundly influenced by the availability of professional health and social care, highlighting the need for further research into the accessibility and conditions of such support [6, 31–36]. Society has both the opportunity and responsibility to support children living with long-term respiratory support and their families, helping them lead healthy and dignified lives as full members of their community [37, 38]. We hypothesised that families with a stronger socioeconomic position (parents with a higher income and/or higher education level, or children born in Sweden with at least one Swedish-born parent) are granted PCA services to a greater extent. Therefore, this study aimed to explore the association between socioeconomic factors and the use of PCAs among children receiving long-term respiratory support.

## Methods

### Study design and settings

This was a retrospective, population-based cohort analysis of children aged 0–18 years living with respiratory support (CPAP, LTMV, HFOT or phrenic pacing) as reported to the Swedish quality registry for respiratory failure (Swedevox) between January 1, 2015, and July 2021. Children were included in the cohort upon starting respiratory support, aged 0–16, and excluded from the cohort upon discontinuing the respiratory support or reaching the age of 18 [5]. Data was sourced from the Swedevox registry and combined with data from Statistics Sweden and the National Register of Municipal Support and Service for Persons with Certain Functional Impairments (LSS registry).

The cohort was assessed, and exclusions were made for those participants in the Swedevox registry who only comprised older data as well as a cut-off of using respiratory support in home environment for less than 30 days.

### Outcomes

Data on the number of approved PCA hours and respite care hours per month were obtained from the LSS registry [39]. Both PCA and respite care hours were summarized into a single outcome variable, as they are used interchangeably by the assistance provider. An average of the allocated hours across all years with recorded data was calculated, resulting in a variable representing the amount of approved PCA hours per month. No data on who applied for PCA were obtained. However, data on PCA and respite care were obtained from the LSS registry, which was sourced from the National Board of Health and Welfare [40].

### Variables

Data on age, sex, type of ventilatory support, connection (tracheotomy/mask), and duration of respiratory support were obtained from the Swedish quality registry for respiratory failure (Swedevox) [13] upon inclusion in the database. Swedevox has been including patients since 1987, but registering children has only been conducted systematically since 2015, hence the starting point of the cohort. The registry has a nationwide multicentre coverage though some geographical blind spots are present [41].

Country of origin was retrieved from the Total Population Registry and dichotomised as born in Sweden with one or two native parents and born in Sweden with two foreign parents or born abroad. The Total Population Registry is kept by Statistics Sweden, recording date of birth, sex and personal relationships for all Swedish citizens since the 1960s [42].

Socioeconomic data regarding disposable household income, parental education levels and parental marital statu**s** were retrieved from the Longitudinal Integrated Database for Health Insurance and Labour Market Studies (LISA) of Statistics Sweden, a database that includes information on income, education employment status and sick leave for all Swedish citizens since 1990 [43]. Disposable household income was index-linked and categorised into tertiles [44], and data on parental education levels were dichotomised as low/ medium (≤ 12 years), and high (> 12 years), corresponding to compulsory/secondary school and postsecondary education (college or university), respectively. Information on marital statu**s** was categorised based on whether the mother lived in a two-parent or single-parent household, while data from the LISA registry were based on the last year before inclusion in the cohort.

### Statistical analysis

The associations between socioeconomic factors and receiving PCA were evaluated using crude and adjusted logistic and linear regression models. To adjust for potential confounding, we included age at the start of treatment as a covariate, as it is associated with an increased need for care and monitoring [36] and is also linked to the age of the parents, which in turn may influence socioeconomic status. Statistical analyses were conducted using SPSS version 28 [45], where p-value < 0.05 was considered statistically significant.

## Results

**Fig. 1:**
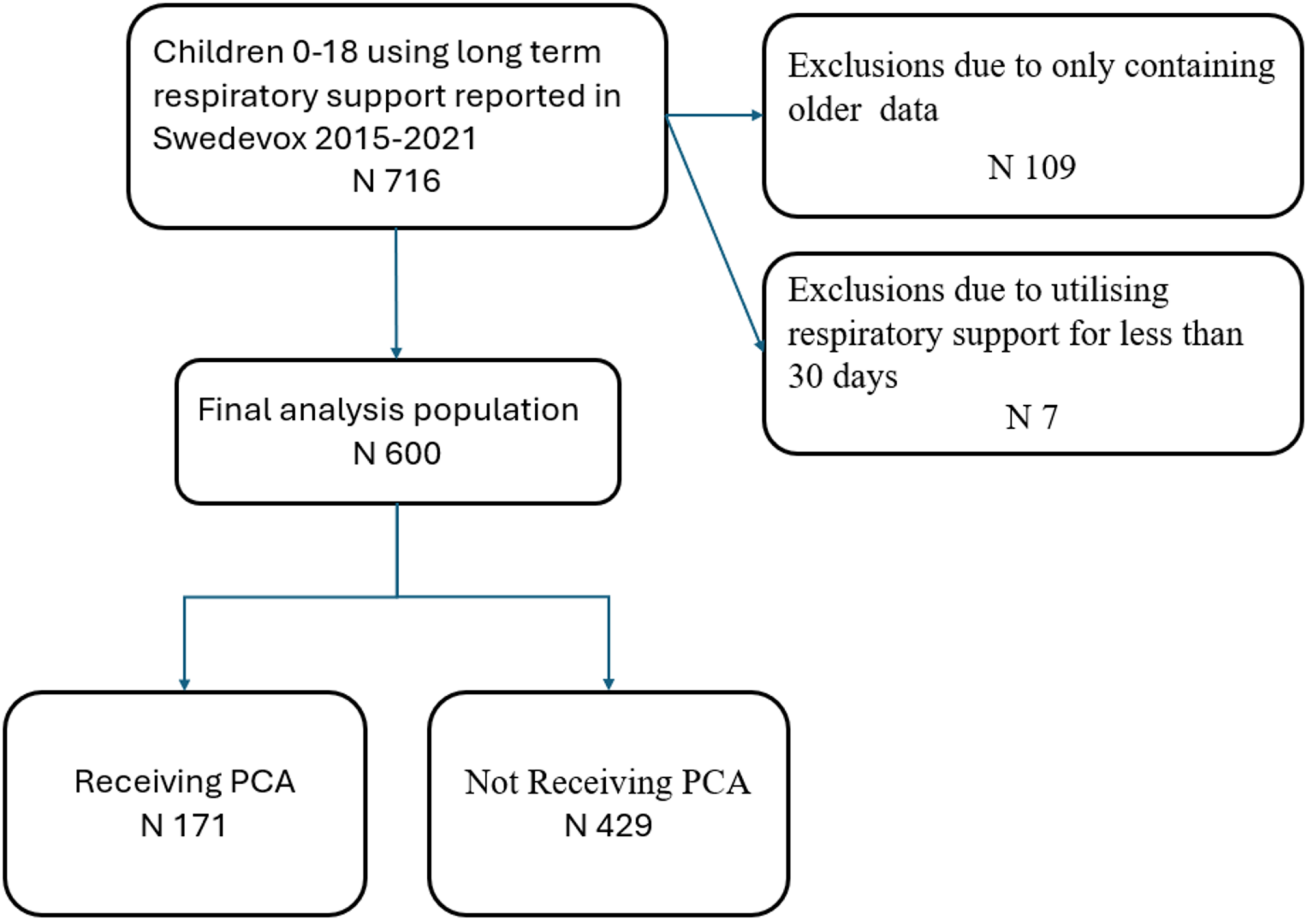
Study flowchart

A total of 600 children were included in the analysis, after excluding those with only older data (*n* = 109) and those who utilised respiratory support for less than 30 days (*n* = 7) (Fig. 1).

Out of the 600 children, 171 (29%) received PCA for a median 235 h/month (interquartile range [IQR] 56–453 h). In the CPAP group (mean age 6.7 ± 5.2 years), 44/199 (22%) received PCA. Among those with home mechanical ventilation (mean age 4.7 ± 5.0 years), 111/348 (32%) received PCA; and among those on other types of respiratory support, such as tracheal cannula only, high-flow oxygen or phrenic nerve pacing (mean age 4.8 ± 4.7 years), 16/53 (30%) received PCA (Table 1). Time using respiratory support in the cohort was a median of 1480 days (IQR: 1992 days). Following changes in legislation, a comparison of children in the cohort reveals an increase in the proportion receiving PCA, rising from 23% in the group up to and including 2019 to 30% in the group after 2019.

**Table 1:** Baseline characteristics

Characteristic	All	Receiving PCA	Not Receiving PCA
No. of children	600	171 (29)	429 (71)
Male sex	340(57)	101(59)	239(56)
Age at start, years	5.4 ± 5.1	5.1 ± 5.0	5.5 ± 5.2
Age at start 0–1 year	216(36)	42(35)	156(36)
Age at start 2–5 years	123(20)	42(25)	81(19)
Age at start 6–10 years	126(21)	34(20)	92(21)
Age at start 11–16 years	135(21)	35(20)	100(23)
Type of respiratory support			
CPAP users	199(33)	44(26)	155(36)
Long-term ventilator support users	348(58)	111(65)	237(55)
Tracheal canula only/ HFOT/phrenic pacing	53(9)	16(9)	37(9)
Tracheotomy users	78(13)	24(14)	54(13)
Country of origin			
Born abroad or born in Sweden with two foreign parents	182(30)	44(26)	138(32)
Born in Sweden with one or two native parents	418(70)	127(74)	291(68)
Disposable Household income (Indexed for 2023) *, **	5698 ± 4918	5648 ± 4546	5826 ± 5768
Tertile 1 (0-4438) (lowest income)	198 (33)	55 (32)	143 (33)
Tertile 2 (4452–6927)	197 (33)	59 (34)	138 (32)
Tertile 3 (6953–71581) (highest income)	197 (33)	53 (31)	144 (34)
Parents’ highest educational level ***			
Medium/low, ≤ 12 y	255(43)	72(42)	183(43)
High, > 12 y	309(52)	93(54)	216(50)
Marital status **			
Two-parent household	469(78)	138(81)	331(77)
One-parent household	129(22)	32(19)	97(23)
Duration of respiratory support (days)	1918 ± 1612	2258 ± 1545	1783 ± 1620

Data are presented as No. (%), mean ± SD. PCA, personal care assistance. CPAP, continuous positive airway pressure. HFOT, High flow oxygen therapy. * Amount presented in hundreds of Swedish kronor (SEK). ** Two missing cases. *** 36 missing cases

Higher family income was not associated with higher odds of receiving PCA (tertile 2, [odds ratio] (1.02; [95% confidence interval] 0.6–1.7) tertile 3, (0.89; 0.5–1.5)) neither was education level (1.08; 0.7–1.6), parents’ marital status (0.91; 0.5–1.6) or country of origin (1.33; 0.9-2.0) (Table 2).

**Table 2:** Logistic regression models with the receiving of personal care assistance services as a dependent variable

Variable	Unadjusted Odds Ratio for the Receiving of PCA (95% CI)	*p* value	Adjusted Odds Ratio for the Receiving of PCA (95% CI)	*p* value
Country of origin				
Born abroad or born in Sweden with two foreign parents	1	…	1	…
Born in Sweden with one or two native parents	0.32 (0.9-2.0)	0.122	1.33 (0.9-2.0)	0.201
Disposable Household income.				
Tertile 1 (lowest income)	1	…	1	…
Tertile 2	1.07 (0.7–1.6)	0.773	1.02 (0.6–1.7)	0.928
Tertile 3 (highest income)	0.92 (0.6–1.4)	0.697	0.89 (0.5–1.5)	0.661
Parents highest educational level				
Medium/low, ≤ 12 y	1	…	1	…
High, > 12 y	1,09 (0,8 − 1,6)	0.629	1.08 (0.7–1.6)	0.701
Marital status				
Two-parent household	1	…	1	…
Single parent household	0,79 (0.5–1.2)	0.304	0.91 (0.5–1.6)	0.726
Age at start continuous variable	0.98 (0.9-1.0	0.356	0.98 (0.9-1.0)	0.389

PCA, personal care assistance

No associations were found between family income (tertile 2([b-Coefficient] 36; [95% confidence interval] -144- 73) tertile 3, (-38; -163- 87)) and monthly allocation of PCA hours, nor were education level (-28; -114- 57), parents’ marital status (95; -29- 220) or country of origin (-7; -107- 92) associated with increased monthly allocation of PCA hours (Table 3).

**Table 3:** Linear regression models with monthly allocation of PCA hours as a dependent variable

Variable	Unadjusted b-Coefficient for Monthly allocation of PCA hours (95% CI)	*p* value	Adjusted b-Coefficient for Monthly allocation of PCA hours (95% CI)	*p* value
Country of origin				
Born abroad or born in Sweden with two foreign parents	1	…	1	…
Born in Sweden with one or two native parents	-3 (-99-93)	0.948	-7 (-107-92)	0.886
Disposable Household income.				
Tertile 1 (lowest income)	1	…	1	…
Tertile 2	-62 (-162-38)	0.226	-36 (-144-73)	0.521
Tertile 3 (highest income)	-117 (-219-(-14))	0.026	-38 (-163-87)	0.554
Parents highest educational level				
Medium/low, ≤ 12 y	1	…	1	…
High, > 12 y	-66 (-152-21)	0.136	-28 (-114-57)	0.517
Marital status				
Two-parent household	1	…	1	…
Single parent household	89 (-18-195)	0.104	95 (-29-220)	0.133
Age at start continuous variable	-18 (-25-(-10))	<0.001	-18 (-27-(-9))	<0.001

PCA, personal care assistance.

## Discussion

The main findings of this longitudinal population-based study including 600 children living with respiratory support in Sweden were that socioeconomic status was not associated with the granting of PCA or the number of allocated hours. The Swedish Social Insurance Agency found that applicants with more substantial financial positions were more likely to be approved on their first application for PCA [46]. However, the findings of this study suggest no significant differences in economic status between families receiving PCA and those not receiving it, distributed over time. According to The Swedish Social Insurance Agency [46], families experiencing economic challenges are more likely to apply for PCA but may face significant obstacles in securing eligibility, possibly due to stringent controls and a complex application process. They also reported that families approved for PCA tend to experience increased income, as they can work as a PCA for their own child.

The present study opposes supposed facts implying that socioeconomic factors influence who receives PCA as stated in articles of debate [15]. Moreover, it shows that socioeconomic status is not reflected in the results of the parents superior advocating skills, which could potentially alter the outcome of interactions with clinicians or bureaucrats [47]. Possible reasons for socioeconomic status not affecting the approval rate could be a well-functioning bureaucratic system, or the consequence of a recently sharpened assessment criteria regarding whom to grant PCA as described in previous studies [15, 18].

Only 29% of individuals in this study received support from PCA. Considering the need for support and studies indicating effects on health-related quality of life as well as substantive sleep deprivation in parents of children living with respiratory support, the number seems low [31, 34, 36, 48]. PCA, which is provided under LSS [14], is free of charge and offers substantial opportunities for individual customisation. In contrast, children who do not qualify for PCA often still have significant care needs, requiring extensive support from both their families and professional caregivers [36]. In Sweden, families whose children do not meet the eligibility criteria for PCA according to LSS [14] may instead be provided support by the municipality under the Social Services Act [49]. However, unlike PCA, support under the Social Services Act is subjected to fees and offers limited possibilities for individual adaptation. Additionally, the overarching goals of these two types of support differ: LSS aims to ensure a good standard of living, whereas the Social Services Act focuses on providing a reasonable standard of living, both in principle and in practice. This distinction in the goals and provisions of care raises important questions about how these differences affect the well-being of children and their families. Further research is needed to explore how variations in the type and quality of support influence family outcomes, caregiver burden, and the overall quality of life for children with substantial care needs. Olin, Dunér and Rauch [15] suggest a potential decline in approval rates for PCA overall and foresee consequences on equality concerns regarding gender, class, or geographical differences for those affected.

Due to legislative changes in 2019, breathing is now considered a basic need under the LSS framework, potentially increasing the care provided to children living with respiratory support. The results indicate a substantial increase in granted PCA, rising from 23% before and including 2019 to 30% in 2020 and onwards. The Swedish Social Insurance Agency [23] analysed the first year following the change and found that 124 individuals aged 0–19, primarily between 0 and 6 years, applied for PCA between November 2019 and June 2020, citing respiratory care as a basic need. Only 42 (34%) were deemed eligible for PCA, and in no more than a handful of cases were respiratory needs the decisive factor. Reasons for denial are not belonging to the LSS target group, not having significant enough needs, and having too advanced respiratory needs.

Health equity means that everyone has an equal and fair opportunity to achieve good health, which requires the elimination of barriers caused by social determinants such as poverty, discrimination, and their consequences [50, 51]. The World Health Organization [52] defines health equity as: “Health equity is achieved when everyone can attain their full potential for health and well-being”. For children requiring respiratory support, access to professional care is often vital involvement and participation in the outside world [6, 36]. Caring ethics, encompassing responsibility, compassion, and a willingness to involve the patient, plays a crucial role in safeguarding these children’s dignity and supporting their development as self-determining individuals [37]. However, to ensure holistic support, caring needs to extend beyond interpersonal interactions and be integrated into the bureaucratic structures that shape healthcare delivery [53]. The results presented in this study reveal no discrepancies in the availability of PCA based on socioeconomic factors, although the sparse number of children approved for PCA highlights whether children who are not approved are provided equal and adequate professional care and assistance.

### Study strengths and limitations

The current study has a number of strengths. First, this patient cohort has a multi-centre, nationwide coverage of children utilising respiratory support in Sweden. In addition, combining data from the Swedevox registry with high-quality socioeconomic information from Statistics Sweden [54] creates a unique and comprehensive database regarding both size and quality [5]. Worth knowing is that, although Swedevox has an overall good national coverage, there are some geographic blind spots in the current dataset. A total of 20 Swedish hospitals have reported children to the cohort; however, one university hospital, two major emergency hospitals, and three regional hospitals are not included. Since all the data in the computations come from mandatory government registries, very few children are lost to follow-up, rendering a data completeness of 93%. Missing data primarily concerns information about parents’ educational level (35 cases). When comparing the included group with the missing ones, significant differences are spotted. The missing group showed significantly higher levels of single-parent households and predominantly comprised children born abroad or with parents born abroad. This group was relatively small, and it had a marginal impact on the overall results. Indeed, when multivariable regression analyses were made without controlling for educational level, none of the results shifted.

Another key limitation is the lack of information on whether families applied for PCA under LSS as we can only analyse who receives PCA and who does not. Another limitation is the small sample sizes in certain groups, which reduced the statistical power and precision of the analyses. Future studies with larger sample sizes are needed.

### Further research

Further studies are needed to explore, through qualitative methods, how the availability or unavailability of PCA impacts the children and families. Additional research should moreover investigate the effects of discrepancies in PCA availability on the outcomes of children utilising respiratory support.

## Conclusions

Among children on long-term respiratory support, 29% were granted PCA, with no apparent association with their socioeconomic status. This suggests that care is provided based on other merits rather than socioeconomic factors. However, the low proportion of children granted PCA raises concerns about whether those who are ineligible receive adequate and equitable support.

## Data Availability

Data can be obtained upon reasonable request. However, the DISCOVERY-P data are not freely accessible due to confidentiality regulations under the Swedish Public Access to Information and Secrecy Act. Access to the data is granted following approval from the Swedish Ethical Review Authority (https://etikprovningsmyndigheten.se) for researchers who satisfy the criteria for accessing confidential information.

## List of abbreviations

CPAP: Continuous positive airway pressure
HFOT: Long-term high-flow oxygen therapy
LTMV: Long-term mechanical ventilation
LISA: Longitudinal Integrated Database for Health Insurance and Labour Market Studies
LSS registry: National Register of Municipal Support and Service for Persons with Certain Functional Impairments
PCA: Personal care assistance
LSS: Swedish Act concerning Support and Service for Persons with Certain Functional Impairments
Swedevox: Swedish quality registry for respiratory failure
